# Peto’s paradox’s relevance is off the scale

**DOI:** 10.18632/aging.206258

**Published:** 2025-05-29

**Authors:** Mirre J.P. Simons

**Affiliations:** 1 School of Biosciences, University of Sheffield, Sheffield, South-Yorkshire S10 2TN, UK

**Keywords:** aging, cancer, evolution

## Abstract

Peto’s paradox is the disconnect between individual risk of cells to develop malignancy and the absence of a strong increase in cancer incidence with body mass across species. Recently small increases of cancer incidence with body mass across species have been interpreted as a falsification of Peto’s paradox. I argue this is a misinterpretation as it is the predicted incredible scale of the increase in cancer incidence with body mass that led to Peto’s paradox. The relevance for the biology of cancer and ageing of Peto’s paradox is off the scale and remains a highly valid paradigm of study.

## INTRODUCTION

The biology of cancer and ageing are strongly intertwined. The risk of the large majority of cancers increases with age [1]. Dietary restriction, perhaps the most consistent and best studied anti-ageing intervention [2], dramatically reduces both cancer incidence and progression [3, 4]. Long-lived species, such as elephants and whales, have evolved different cellular anticancer mechanisms [5, 6]. Interestingly, other long-lived animals have evolved very different mechanisms than our own. For example, in comparison bats evolved limited cell-intrinsic barriers to prevent excessive proliferation [7, 8]. The field of comparative biology into ageing and cancer was given a strong impetus when Peto identified that humans have substantially more cells than mice, but do not have substantially larger incidence of cancer [9, 10]. Peto’s paradox has subsequently been loosely interpreted as representing the lack of correlation between a species’ body mass and their cancer incidence [11–13]. Some of these interpretations go as far as rejecting Peto’s paradox based on their recent finding of a small increase of cancer prevalence with species’ body mass [14]. Although another recent paper gives a more nuanced interpretation of a similar finding [15].

**Figure 1:**
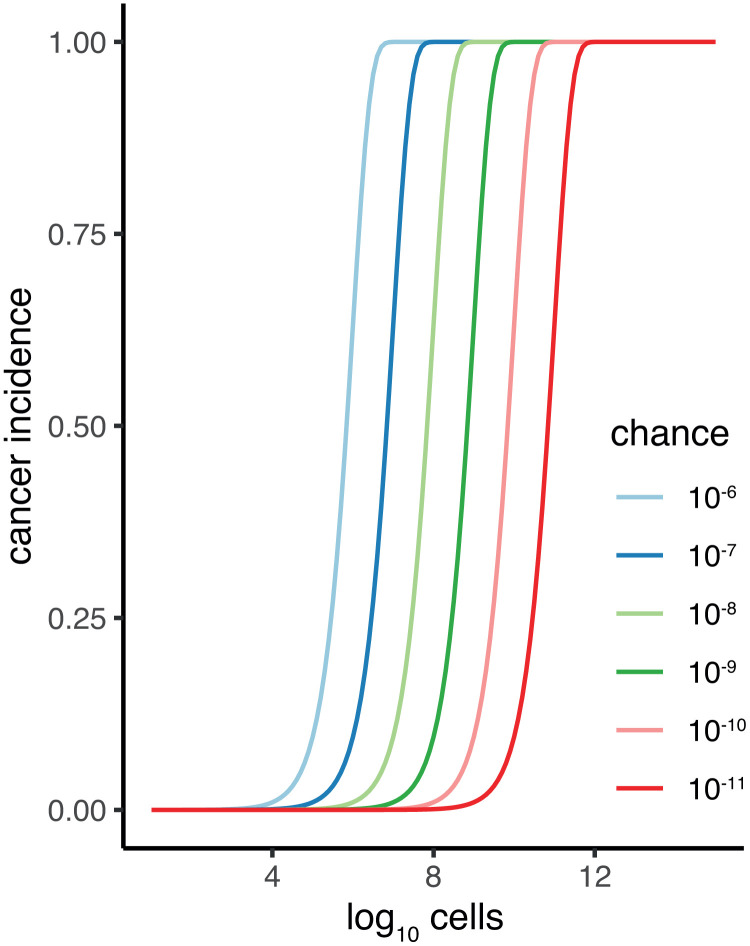
**Cancer incidence plotted against total somatic cell count (*C*) of an organism for difference *p* (chance) of malignancy per cell.** Calculation of cancer incidence is the chance of at least one cell becoming malignant: P_maligancy_ = 1-(1-*p*)^C^.

The original identification of Peto’s work presenting a paradox [9, 10, 15] did so by pointing to the lack of a strong positive correlation between cancer incidence and a species’ body mass [10]. The problem posed by Peto’s paradox is that if individual cells have the same risk per time to acquire malignancy, then bigger organisms that are composed of more cells should have much higher cancer incidence. Malignancy is predicted to increase dramatically with body size if only one damaged cell is required for a tumour to develop. Detailed modelling and predictions have been made previously [16, 17]. For illustration purposes imagine a chance of one in a million for a cell to develop malignancy per a given time. Predicted cancer incidence is limited for species with low cell count, increases dramatically for species with 10,000 cells, and saturates with all individuals getting cancer at around three million cells (Figure 1). Note, this discrepancy is even bigger as body mass co-evolves strongly with lifespan, over a scale of over hundred-fold in vertebrates [5, 18]. Bigger animals thus both have more cells at risk but also have a longer time to accrue somatic damage that leads to malignancy.

**Figure 2:**
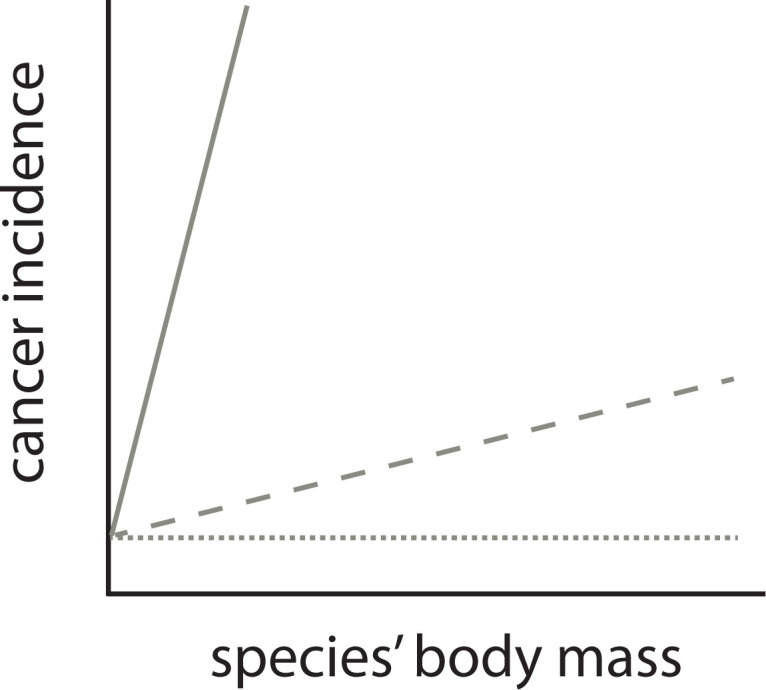
**The relationship between a species’ body mass and cancer incidence is not predicted to be flat (dotted line) by Peto’s paradox.** It is the predicted dramatic relationship with body mass that is the null hypothesis for Peto’s paradox (solid line). A comparative relationship (dashed) that has a shallower slope than this null hypothesis is no proof against the validity of Peto’s paradox. Note, however, that the exact shape of null prediction of the relationship between a species’ body mass and cancer incidence is not known, it is however predicted to be dramatic (as in Figure 1).

The observation that cancer incidence increases with body mass [15], therefore, in itself does not resolve Peto’s paradox, contrary to the conclusions of Butler et al. [14]. Instead, it is the magnitude of increase in cancer incidence scaled by body mass which is critical. The null hypothesis of Peto’s paradox of the relationship between a species’ body mass and cancer incidence is not that there is no relationship. The paradox is resolved when cancer incidence would increase dramatically with a species’ body mass (Figure 2). Perhaps fitting with this interpretation mistake is that the incidence metric reported in the main text by Butler et al. is without clear units, due to the way count observation data are modelled. However clearly ~2.5% to ~7% in cancer incidence on a linear scale (Fig S13 in Butler et al. 2025) across 8 (natural log) powers of magnitude in body mass is a small effect and this does not come near the scale of the effect of Peto’s paradox (Figure 1). The increase reported in Butler et al. is similar (1.30% slope on log_10_ scale) to the slopes reported in Compton et al. (0.65% and 2.9% when corrected for gestation time [15]). The future of comparative oncology should not be aimed to refute Peto’s paradox [14] but as Compton et al. argue should be aimed at explaining variation in rates of malignancy across animal species. As in human epidemiology of cancer, assessing incidence is data hungry. The different levels of accuracy due to sample size and different levels of bias, may still limit this quest to the species at the extreme ends of this variation.

Another explanation for Peto’s paradox is that we do not understand the underlying variables well enough to make an accurate null hypothesis of how cancer incidence should scale with age [19]. Still, the original simplicity of Peto’s paradox is its strength. Any adaptation that co-evolves with body mass that changes the susceptibility to cancer is of fundamental and possibly ultimately clinical relevance. Especially relevant and probably clinically relevant is how different species’ tissue microenvironment constraints or facilitates malignant growth. Indeed, it is becoming increasingly recognised that somatic mutation is not the sole explanation for carcinogenesis [20]. It is without doubt that Peto’s paradox holds. Understanding how large-bodied organisms have evolved resistance to cancer, especially in exceptionally large mammals has the potential to reveal mechanisms that can be harnessed in oncology and biology of ageing research [5].
